# Determinant factors for the occurrence of tuberculosis after initiation of antiretroviral treatment among adult patients living with HIV at Dessie Referral Hospital, South Wollo, Northeast Ethiopia, 2020. A case-control study

**DOI:** 10.1371/journal.pone.0248490

**Published:** 2021-03-16

**Authors:** Mehd Abdu, Yeshimebet Ali, Samuel Anteneh, Mohammed Yesuf, Adane Birhanu, Salih Mohamed, Adem Hussien

**Affiliations:** 1 Department of Nursing, College of Medicine and Health Sciences, Mizan-Tepi University, Mizan Teferi, Ethiopia; 2 Department of Public Health, College of Medicine and Health Sciences, Wollo University, Dessie, Ethiopia; 3 Department of Adult Health Nursing, College of Medicine and Health Sciences, Wollo University, Dessie, Ethiopia; 4 Department of Nursing, College of Medicine and Health Sciences, Debre Tabor University, Debre Tabor, Ethiopia; 5 Department of Anesthesia, College of Medicine and Health Science, Wollo University, Dessie, Ethiopia; 1. IRCCS Neuromed 2. Doctors with Africa CUAMM, ITALY

## Abstract

**Introduction:**

Globally, tuberculosis takes the first rank for the ill-health of people living with HIV/AIDS. Despite the favorable outcome of antiretroviral therapy, the risk of tuberculosis remains higher among HIV patients. This obliges to identify factors for its occurrence and further prevention of drug-resistant tuberculosis. There is a contradiction between different studies and studies conducted in Ethiopia studied poorly the association between adherence to antiretroviral therapy and viral load with tuberculosis. Studies conducted in the study area were limited to cross-sectional study design. Therefore, this study claimed to identify factors determining the occurrence of tuberculosis after initiation of antiretroviral therapy.

**Methods:**

This study was conducted at Dessie Referral Hospital by using a case-control study design on a sample of 565 with a control: case ratio of 3:1. Participants from controls were selected by systematic random sampling and from cases by consecutive random sampling. The data were collected by interviewing through structured questionnaires and from the medical record. The data were entered into Epi data version 3.1. In the multivariable analysis, variables with a P-value of ≤0.05 were anticipated as independent determinant factors.

**Result:**

Patients without separate kitchen (AOR: 3.547, 95% CI: 2.137, 5.888), having opportunistic infection (AOR: 3.728, 95% CI: 2.058, 6.753), CD4 count of <350 cells/mm3 (AOR: 3.383, 95% CI: 1.520, 7.528), baseline WHO stage III (AOR: 3.321, 95% CI: 1.688, 6.534) or IV (AOR: 2.900, 95% CI: 1.251, 6.722), don’t taking IPT (AOR: 3.701, 95% CI: 2.228, 6.147) and those who were poorly adherent (AOR: 2.626, 95% CI: 1.272, 5.423) or moderately adherent (AOR: 3.455, 95% CI: 1.885, 6.335) to anti-retroviral therapy were more likely to develop tuberculosis after anti-retroviral therapy initiation.

**Conclusion:**

Poor housing conditions, having an opportunistic infection, low CD4 count, starting ART at the advanced HIV stage, don’t take IPT, and being poorly adherent to antiretroviral therapy were associated with the occurrence of TB after initiation of ART. The institution should screen for TB as early as possible and strictly follow their drug adherence.

## Introduction

Tuberculosis (TB) is an extremely contagious bacterial infection caused by aerobic bacteria called mycobacterium tuberculosis. Despite commonly affecting the lung, TB can also involve other body parts particularly among people living with Human Immunodeficiency Virus (HIV), called extra-pulmonary TB [1]. HIV infection has been identified as the major predisposing factor for being infected by TB. Being infected by both HIV/AIDS and TB is called TB/HIV co-infection [1,2].

The Ethiopian Federal Ministry of Health recommended the use of isoniazid for eligible HIV patients to intercept the reactivation of latent TB to active TB disease. It should be administered for six months starting at enrollment to HIV care after ruling out active TB disease [3]. Globally, HIV continues to take a leading role in the ill-health of human beings. Globally, about 36.7 million patients were living with HIV/AIDS and 2.1 million people became newly infected in 2015. Sub-Saharan Africa accounts for the larger proportion with 25.6 million patients living with the virus. In Ethiopia, the national prevalence of HIV for the year 2017 was 1.16% [4].

Antiretroviral therapy (ART) is treating a patient with HIV medicine [2]. Antiretroviral therapy should be initiated for every individual immediately after the confirmation of HIV diagnosis regardless of their WHO clinical stage and CD_4_ count [3].

Early identification of TB among patients living with HIV through careful evaluation of clinical manifestations and using proper investigation improves survival, quality of life, and reduce transmission within the health care setting as well as in the community. The Anti-TB drug should be initiated immediately after the confirmation of TB. ART should be initiated within two weeks of Anti-TB initiation depending on the clinical conditions of the patient [5].

Tuberculosis is among the major disease burdens worldwide. According to the world health organization (WHO) report, in 2019, about 10 million people develop TB [6]. Globally, about 1.7 billion people became infected with TB; 5–10% develop active TB disease in their life time [7]. Reactivation of latent TB is higher among people living with HIV [2]. Africa is the second TB burden region (25%) next to south-east Asia (44%) [6]. However, the incidence of TB among patients living with HIV is highest in Africa region [7].

A decrease in the incidence of TB is reported among people living with HIV (PLWHIV) by the initiation of potent ART [8]. Despite the favorable effects of ART, the risk to develop TB remains higher among PLWHIV than the general population [1]. This obliges to identify factors for its occurrence and further prevention of MDR TB. According to a study conducted in Northeast Ethiopia, 30.3% of patients who were on ART developed active TB disease [9]. It takes the first rank for morbidity and mortality among PLWHIV worldwide; about 8.6% of the total TB patients were also co-infected with HIV/AIDS. Globally in 2018, there were about 251,000 deaths from TB among PLWHIV, which accounts for 33% of total deaths associated with HIV which is much higher than the case fatality rate expected by WHO, which is ≤5% [4].

Different studies identified that advanced WHO clinical stages while initiating ART, co-morbid conditions, low hemoglobin level, IPT use, and low CD_4_ count contribute to the occurrence of opportunistic infections including tuberculosis after initiation of ART [10,11].

People living with HIV (PLWHIV) who have active TB disease were prone to several adverse outcomes. They have the risk of increased mortality, developing AIDS Defining Event, Loss to Follow Up rate, and developing Multiple Drug-Resistant Tuberculosis (MDR TB) [12]. One study done in Ethiopia showed that 79.8% of patients with MDR TB were HIV positive [13].

Sustainable development goals and end TB strategy of WHO includes the reduction of the total TB burden as one element of their target [7]. Ethiopia has also developed a five-year strategic plan to maintain a satisfactory path for ending AIDS by 2030 [4]. The recent guideline recommended ART initiation for all HIV-positive clients irrespective of CD_4_ count or WHO stage. Despite the importance of early initiation, immunological or clinical improvement and viral suppression will not be achieved without strict adherence to ART [3,14].

There are contradictions between studies regarding factors determining the development of TB after ART initiation. Studies conducted in Ethiopia also poorly studied the association between adherence and viral load with the development of TB. A cross-sectional study design was used by the studies conducted in the study area. Hence, this study intended to provide information about determinant factors to the occurrence of tuberculosis after initiation of ART among PLWHIV having follow-up at Dessie Referral Hospital with a case-control study design.

## Methods and materials

### Study design and period

An institution-based unmatched case-control study design was employed to identify factors determining the occurrence of TB after initiation of ART among patients living with HIV/AIDS at Dessie referral hospital in Dessie town which is located 401 Km away from Addis Ababa. The study was conducted from 6 March 2020 to 12 April 2020.

### The study participants

All adult patients on ART who were taking the full course of anti-tuberculosis medication or newly diagnosed for tuberculosis by the clinician were the source population for cases and those on ART but not taking the full course of anti-TB medication or not diagnosed as having TB were the source population for controls. Randomly selected adult patients who were available during the data collection period were the study population. Those who loss to follow up and patients who came to start ART were excluded.

### Sample size determination and sampling technique

The sample size was calculated for each exposure variables considered as statistically significant by the selected study in the literature by using EpiInfo version: 7.2.3.1 software by considering the Confidence level of 95%, power of 80%, and control to case ratio of 3:1. The variable which results in a larger sample (marital status) was selected. The sample size became 538 [15]. By adding a 5% non-response rate, the sample size became 565; 424 controls and 141 cases [15].

It was expected that 1188 controls and 176 cases will attend the ART clinic. Among them, 424 Controls were selected by systematic random sampling and 141 cases were selected by consecutive random sampling.

### Data collection procedures

Data was collected by interviewing through structured interviewer-administered questionnaires and reviewing the medical record of the patient. Clinical data (CD_4_ cell count, hemoglobin level, BMI, baseline WHO clinical stage, IPT use, diagnosis of opportunistic infections, and TB) was obtained from the medical record of the patient. Other information (Socio-demographic characteristics, host and environmental factors, and adherence to ART) was gathered by interviewing the client through a structured questionnaire. First, information to be taken through the interview was taken from the patient. Then the remaining information was taken from the chart after the patient exits.

### Measurements

The outcome variable (TB diagnosis) was done based on the Ethiopian national TB guideline [16]. The structured questionnaire was adopted after reviewing different pieces of literature [9,15,17] and reviewed by the experts to check for face validity. Adherence to ART was measured by a validated eight-item Morisky medication adherence scale (MMAS-8). The scale contains eight questions. The first 7 are dichotomous (Yes or No) in which point 0 is given for response Yes and 1 for No. The 8^th^ question contains 5 possible responses (Never/rarely, Once in a while, Sometimes, Usually, and All the time); 1 point is given for Never/Rarely, and 0 for others. The total score is calculated by adding all individual MMAS-8 question scores. The scale has Cronbach’s alpha value of 0.83 [18].

### Operational definitions

**Smear positive pulmonary tuberculosis**: at least one sputum smear examination positive for Acid Fast Bacilli (AFB) by direct microscopy. **Smear-negative pulmonary tuberculosis**: sputum specimens negative for AFB and radiographical abnormalities were consistent with active TB and decision by a clinician to treat with a full course of anti-tuberculosis chemotherapy. **Extra-pulmonary tuberculosis**: Clinically consistent with active Extrapulmonary TB and bacteriologically confirmed by AFB of one specimen from an extra-pulmonary site for mycobacterium tuberculosis and a decision by a clinician to treat with a full course of anti-TB chemotherapy [16].

**Current Smoker:** smoking at least 1 cigarette per day in the past 30 days [19].

**Current Khat chewer:** a person who chews leaves of the plant Khat in any amount in the past 30 days [20].

**Alcohol drinker**: individuals who had a history of alcohol use in the past 30 days [21].

**Adherence to ART:** An individual with a score of 0 to 5 by the Morisky Medication Adherence Scale is considered as non-adherent, 6 to 7 as medium adherent, and 8 high adherents [22].

**Functional status:** the patient is categorized as **working** if the patient can perform his/her usual works in or out of the house, **Ambulatory** if he/she can perform activities of daily living, and **Bedridden** if not able to perform activities of daily living [15].

### Data quality assurance

A pretest was conducted on 5% of the sample size at Wogide primary Hospital to check whether the questions to be interviewed are understandable by the respondents and to check the reliability of the questionnaires. First, the questionnaire was prepared in English, translated to Amharic, and then back-translated to English for consistency. Data was collected by 4 data collectors; 2 BSc nurses and 2 BSc public health holders who have work experience at ART clinic. The data collectors were supervised by a public health professional who is BSc and MPH fellow. Data collectors took training for two days. Appropriate and comprehensive instructions were given for the data collectors by the principal investigator to ensure the quality of the data. The filled questionnaire was checked on the day of its collection for completeness.

### Data processing and analysis

The collected data were checked for completeness and consistency and then each questionnaire was coded and entered into Epi Data version 3.1 software and exported to SPSS version 25.0 for analysis. Hosmer-Lemshow test was checked to evaluate whether the assumption for binary logistic regression is fulfilled or not and to evaluate model fitness in which p-value > 0.05 indicates that the assumption is fulfilled and the model is fitted with data. A bivariable binary logistic regression was done for each explanatory variable with the dependent variable. Variables with a p-value of ≤ 0.25 in a bivariable analysis were entered to multivariable binary logistic regression [15]. The result was described and expressed by using tables, graphs, and narrative descriptions. Variables with a P-value of ≤ 0.05 in the multivariable binary logistic regression analysis were considered a measure of a statistically significant determinant.

### Ethical consideration

Ethical clearance and approval letter were obtained to conduct this research from Wollo University College of medicine and health science research and ethical review committee. Permission was requested from Dessie Referral Hospital before starting data collection. The purpose and objective of the study were explained to the respondents selected for the interview. The participants were informed that the privacy and confidentiality of their information are respected. The participants were also informed that the research doesn’t have short term financial and healthcare benefit and there is no physical harm, social stigma, psychological trauma, and economic loss. The participants were informed that they are not enforced to participate in the study and can discontinue at any time of data collection. Voluntary written informed consent was obtained from the participants based on the consent form designed for this study.

## Result

### Socio-demographic characteristics of study participants

Out of 565 samples selected, 556 of them responded (417 controls and 139 cases). The response rate became 98.6% for cases and 98.3% for controls. The overall response rate becomes 98.4%. The mean ages for overall respondents were 41.64 years with a standard deviation of 11.701. About two-thirds of the respondents from cases, 254(63.3%), and 88(60.9%) from controls were females. More than half of the controls, 72(61.9%), and cases 258(51.8%) were married. More than half of controls, 75(58.5%), and cases, 244(54%), were Muslim. The larger proportion of controls, 118(28.3%), were uneducated; whereas a larger proportion of cases, 39(28.1%), were completed secondary education. About 164(39.3%) of controls and 57(41%) of cases were self-employed. About half of the controls, 217(52%), and 71(51.1%) of cases had a family size of 3–4 within the household **(Table 1).**

**Table 1:** Socio-demographic characteristics of HIV patients at Dessie Referral Hospital, 2020(n = 556).

Variables	Categories	Cases		Controls		Total	
		N	%	N	%	N	%
**Age (mean** **±** **SD)**		43.28 ± 12.414		41.10 ± 11.417		41.64 ± 11.701	
**Gender**	Male	51	36.7	163	39.1	214	38.5
	Female	88	63.3	254	60.9	342	61.5
**Marital status**	Single	21	15.1	50	12.0	71	12.8
	Married	72	51.8	258	61.9	330	59.3
	Divorced	24	17.3	52	12.4	76	13.7
	Widowed	22	15.8	57	13.7	79	14.2
**Religion**	Orthodox	54	38.8	151	36.2	205	36.9
	Muslim	75	54.0	244	58.5	319	57.4
	Protestant	7	5.0	21	5.1	28	5.0
	Others	3	2.2	1	0.2	4	0.7
**Educational status**	Uneducated	38	27.3	118	28.2	156	28.0
	Primary school	37	26.6	112	26.9	149	26.8
	Secondary school	39	28.1	112	26.9	151	27.2
	Tertiary education	25	18.0	75	18.0	100	18.0
**Occupation**	Self-employed	57	41	164	39.3	221	39.7
	Gov’t employed	14	10.1	62	14.9	76	13.7
	Unemployed	6	4.3	26	6.2	32	5.8
	Housewife	23	16.5	74	17.7	97	17.4
	Farmer	28	20.1	74	17.7	102	18.3
	Student	11	8.0	17	4.1	28	5.0
**Family size**	≤2	20	14.4	58	13.9	78	14.0
	3–4	71	51.1	217	52.0	288	51.8
	≥5	48	34.5	142	34.1	190	34.2
**Residence**	Urban	71	51.1	259	62.1	330	59.4
	Rural	68	48.9	158	37.9	226	40.6

Gov’t = Government.

### Host and environmental characteristics of study participants

**Fig 1:**
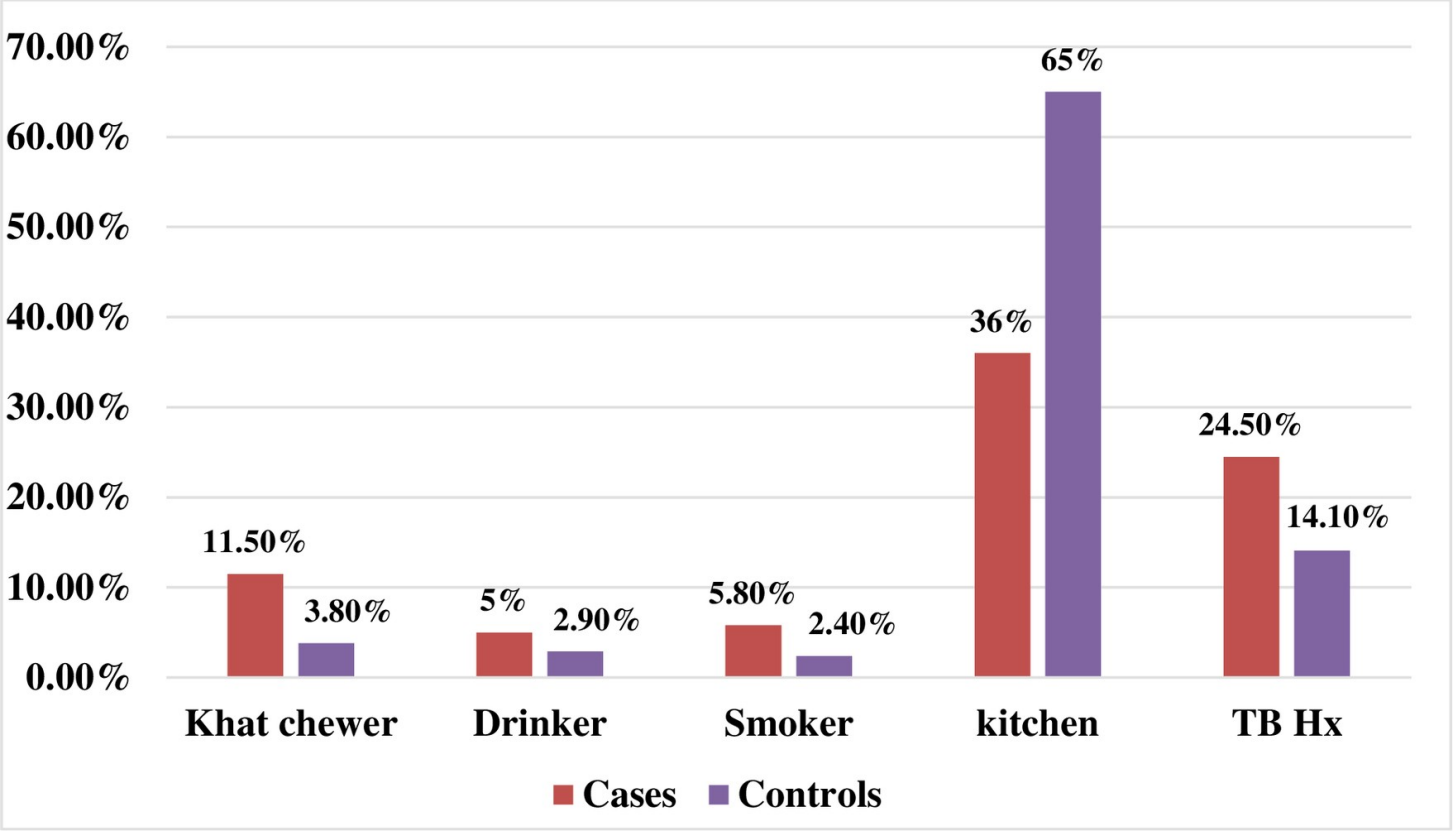
Host and environmental characteristics of HIV patients at Dessie Referral Hospital, 2020(n = 556).

Only a small proportion of cases, 16(11.5%), and controls, 16(3.8%), were chat chewer. The majority of cases, 132(95%), and controls, 405(97.1%), were not alcohol drinkers. A larger proportion of cases, 131(94.2%), and controls, 407(97.6%), were not a smoker. Slightly more than one-third of cases, 50(36%), had a kitchen separated from a living room. However, the majority of controls, 271(65%), had a kitchen separated from a living room. About one-quarter of cases, 34(24.5%), and 59(14.1%) of controls had a previous history of TB **(Fig 1)**.

### Clinical and immunological characteristics of study participants

The majority of the respondents, 461(82.9%), were working in their baseline functional status. A small proportion of the respondents, 35(6.3%), had a history of DM. More than one-third of cases, 55(39.6%) and 49(11.8%) of controls had an opportunistic infection. The mean duration of ART of the respondents was 96.45 months with an SD of 37.62. A larger proportion of cases, 43(30.9%), were in WHO stage III while starting ART whereas a larger proportion of controls, 180(43.2%), were in WHO stage I while starting ART **(Table 2)**.

**Table 2:** Clinical and immunological characteristics of HIV patients at Dessie Referral Hospital, 2020(n = 556).

Variables	Categories	Cases		Controls		Total	
		N	%	N	%	N	%
**Functional status**	Working	97	69.8	364	87.3	461	82.9
	Ambulatory	29	20.9	40	9.6	69	12.4
	Bedridden	13	9.3	13	3.1	26	4.7
**DM**	Yes	22	15.8	13	3.1	35	6.3
	No	117	84.2	404	96.9	521	93.7
**Having OIs**	Yes	55	39.6	49	11.8	104	18.7
	No	84	60.4	368	88.2	452	81.3
**on ART (mean** **±** **SD)**^**a**^		90.74 ± 38.206		98.35±37.275		96.45 ± 37.62	
**WHO clinical stage**	Stage I	30	21.6	180	43.2	210	37.8
	Stage II	37	26.6	140	33.6	177	31.8
	Stage III	43	30.9	69	16.5	112	20.1
	Stage IV	29	20.9	28	6.7	57	10.3
**CD**_**4**_ **count(mean** **±** **SD)**^**b**^		416.14±330.916		622.34±307.91		570.32±326.126	
**BMI (mean** **±** **SD)**^**c**^		18.066 ± 1.4223		19.062±2.0191		18.813 ± 1.9352	
**Hgb (mean** **±** **SD)**^**d**^		12.154 ± 0.8177		12.434±1.0798		12.36 ± 1.026	
**IPT use**	Yes	58	41.7	338	81.1	396	71.2
	No	81	58.3	79	18.9	160	28.8
**Adherence to ART**	Poor	22	15.8	44	10.6	66	11.9
	Moderate	41	29.5	59	14.1	100	18.0
	Good	76	54.7	314	75.3	390	70.1

^a^ = measured in month

^b^ = measured in cells/mm^3^.

^c^ = measured in kg/m^2^

^d^ = measured in g/dl.

### ART adherence status of the study participants

**Fig 2:**
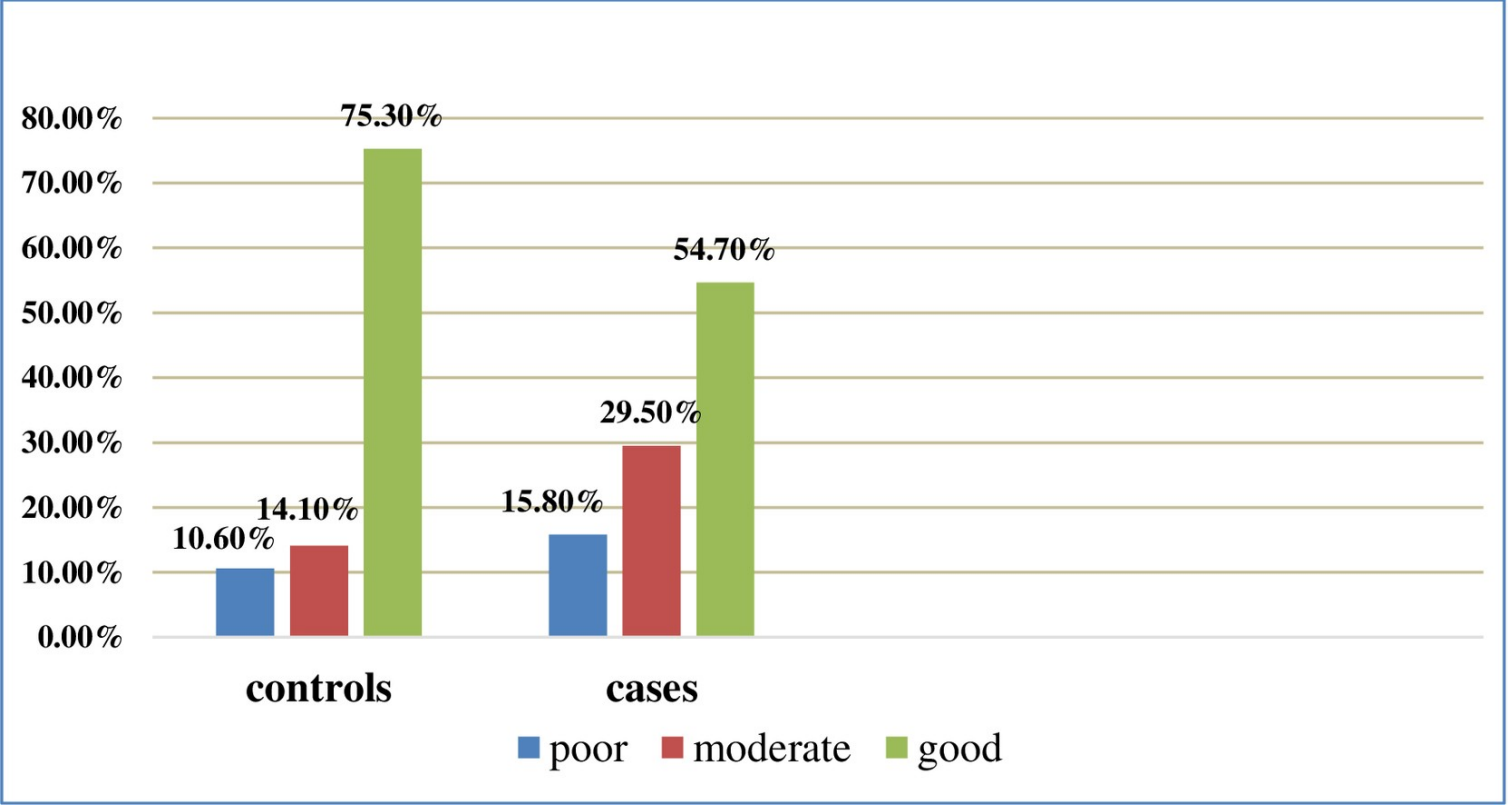
ART adherence status of HIV patients at Dessie Referral Hospital, 2020(n = 556).

About three fourth of controls and more than half of cases had good adherence to the prescribed antiretroviral medicine **(Fig 2).**

### Clinical presentation of cases

**Fig 3:**
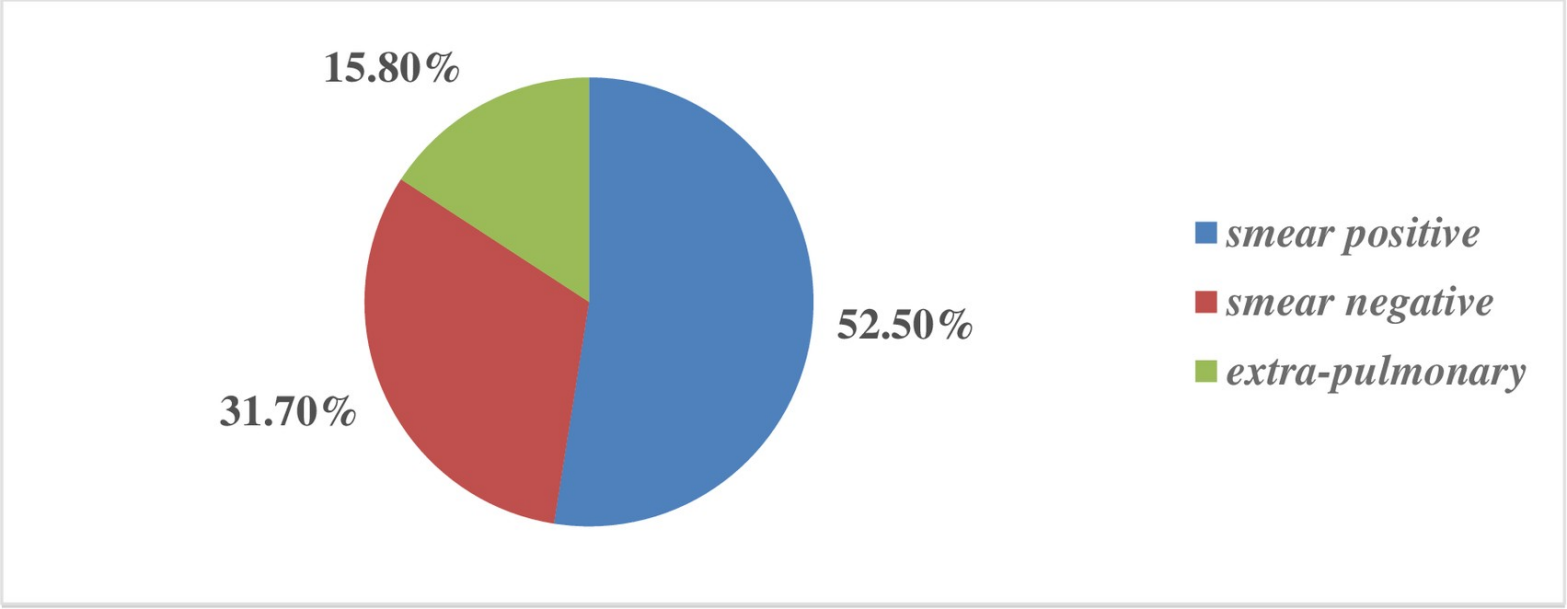
Clinical presentations of TB-HIV co-infected patients at Dessie Referral Hospital, 2020(n = 556).

A larger proportion, 73 (52.5%), of patients presented with TB were diagnosed as having smear-positive pulmonary TB **(Fig 3)**.

### Factors associated with the occurrence of TB after initiation of ART

Variables with a p-value of ≤ 0.25 in the bivariable binary logistic regression analysis were selected as eligible for multi-variable binary logistic regression analysis. These variables include- Socio-demographic factors: marital status, occupation, residence, monthly income; host and environmental factors: chat chewing, alcohol drinking, smoking, having separate kitchen, previous TB; clinical and immunological factors: functional status, DM, OIs, baseline WHO stage, CD4 count, viral load, BMI, IPT use, and adherence to ART. Multivariable binary logistic regression analysis was done to identify factors that independently determine the occurrence of TB after initiation of ART by controlling the effect of other factors. Some variables became independent predictors of TB after initiation of ART after controlling for the other factors. Patients who do not have a separate kitchen were more likely to develop TB (AOR = 3.547 95% CI = 2.137, 5.888 P = 0.000). Patients having an opportunistic infection were more likely to develop TB (AOR = 3.728 95% CI = 2.058, 6.753 P = 0.000). Patients with a baseline WHO stage III (AOR = 3.321 95% CI = 1.688, 6.534 P = 0.001) OR IV (AOR = 2.900 95% CI = 1.251, 6.722 P = 0.013) were more likely to develop TB as compared with those with WHO stage I. patients having a CD4 count of 201–349 cells/mm3 (AOR = 3.383 95% CI = 1.520, 7.528 P = 0.003) were more likely to develop TB as compared with those having a CD4 count of ≥350 cells/mm3. Patients who did not take IPT were more likely to develop TB (AOR = 3.701 95% CI = 2.228, 6.147 P = 0.000). Patients who are poorly adherent (AOR = 2.626 95% CI = 1.272, 5.423 P = 0.009) or moderately adherent (AOR = 3.455 95% CI = 1.885, 6.335 P = 0.000) to ART were more likely to develop TB as compared with those who are good adherent. Marital status, occupational status, income status, residence, chat chewing, smoking, alcohol drinking, previous TB, functional status, history of DM, viral load, and BMI were not statistically associated with the occurrence of TB after ART in the multivariable binary logistic regression analysis **(Table 3).**

**Table 3:** Factors determining the occurrence of TB after ART initiation among HIV patients at Dessie Referral Hospital, 2020(n = 556).

Variables	Categories	Cases	Controls	COR (95% CI)	P-value	AOR (95% CI)	P-value
Monthly income (birr)	< 1000	19	97	1			
	1000–2000	71	185	1.959 (1.116, 3.439)	.019		
	≥ 2000	49	135	1.853 (1.027, 3.344)	.041		
Residence	Urban	71	259	1			
	Rural	68	158	1.570 (1.066, 2.312)	.022		
Khat	Yes	16	16	3.260 (1.584, 6.710)	.001		
	No	123	401	1			
Separate kitchen	Yes	50	271	1		1	
	No	89	146	3.304 (2.213, 4.932)	.000	3.547 (2.137, 5.888)	.000*
Hx of TB	Yes	34	59	1.965 (1.222, 3.159)	.005		
	No	105	358	1			
Functional status	Working	97	364	1			
	Ambulatory	29	40	2.721 (1.605, 4.612)	.000		
	Bedridden	13	13	3.753 (1.685, 8.358)	.001		
DM	Yes	22	13	5.844 (2.856, 11.955)	.000		
	No	117	404	1			
OIs	Yes	55	49	4.917 (3.129, 7.728)	.000	3.728 (2.058, 6.753)	.000*
	No	84	368	1		1	
Baseline WHO stage	Stage I	30	180	1		1	
	Stage II	37	140	1.586 (0.934, 2.693)	.088	1.503 (0.796, 2.839)	.209
	Stage III	43	69	3.739 (2.173, 6.433)	.000	3.321 (1.688, 6.534)	.001*
	Stage IV	29	28	6.214 (3.253, 11.872)	.000	2.900 (1.251, 6.722)	.013*
CD4 count/cells/mm^3^	≤200	42	39	5.836 (3.494, 9.750)	.000	1.797 (0.776, 4.161)	.171
	201–349	34	34	5.419 (3.136, 9.366)	.000	3.383 (1.520, 7.528)	.003*
	≥350	62	336	1		1	
Viral load (copies/ml)	< 1000	57	326	1			
	≥ 1000	80	83	5.513 (3.635, 8.361)	.000		
IPT use	Yes	58	338	1		1	
	No	81	79	5.975 (3.939, 9.064)	.000	3.701 (2.228, 6.147)	.000*
Adherence to ART	Poor	22	44	2.066 (1.168, 3.652)	.013	2.626 (1.272, 5.423)	.009*
	Moderate	41	59	2.871 (1.793, 4.597)	.000	3.455 (1.885, 6.335)	.000*
	Good	76	314	1		1	

* Statistically significant (p-value ≤0.05), Hx: History.

## Discussion

This study has identified some environmental and clinical predictors for the occurrence of TB after ART initiation among patients living with HIV. Patients who don’t have a separate kitchen, those with baseline WHO stage III or IV, a CD4 count of < 350 cells/mm3, those who developed an opportunistic infection, those who are poorly adherent to the prescribed antiretroviral therapy, and those who didn’t take IPT were at higher risk to develop TB after ART initiation.

According to this study, having a separate kitchen has a statistically significant association with the occurrence of TB in which patients who don’t have a separate kitchen were more likely to develop TB (AOR: 3.547 95% CI: 2.137, 5.888), which is consistent with a case-control study conducted at Addis Ababa and western Ethiopia in which patients who have a separate kitchen were less likely to develop TB after ART initiation [23,24]. This may be due to the effect of indoor air pollution which occurs as the individual cooks within the house. Adequate ventilation, either natural or artificial, is one of the recommendations by FMoH guidelines for the prevention of TB among patients with HIV [3].

This study identified that patients who developed opportunistic infections were more likely to develop TB after ART initiation (AOR: 3.728 95% CI: 2.058, 6.753) which is similar to a retrospective cohort study conducted at Debre-Markos referral Hospital and a case-control study conducted at Addis Ababa [23,25]. This may be explained by the occurrence of opportunistic infection indicates a decreased immune function of the patient. As supported by different studies, the occurrence of OIs not only determines the development of TB among HIV patients but also increases mortality among TB/HIV co-infected patients [26]. National strategies recommended chemoprophylaxis and starting ART for the prevention of the occurrence of opportunistic infections [27].

This study identified baseline WHO stage as a determinant factor for the occurrence of TB after ART initiation in which patients with baseline WHO stage III (AOR: 3.321 95% CI: 1.688, 6.534) or IV(AOR: 2.900 95% CI: 1.251, 6.722) were more likely to develop TB after ART as compared with those patients with baseline WHO stage I, which is consistent with a retrospective study conducted in Cameroon which showed that patients with WHO stage III or IV were at higher risk for developing TB as compared with those with stage I or II [28]. It is also similar to a cross-sectional study conducted in the Amhara region in which patients with WHO stage I and II were less likely to develop TB as compared with those patients with WHO stage IV [15]. This implies the effect of a compromised immune system during initiation of ART for the development of TB after initiation of ART. The study is supported by the national guideline of Ethiopia for comprehensive HIV prevention, care, and treatment in which starting ART early regardless of WHO stage has a crucial effect for clinical improvement and improved survival. As described by the guideline, early initiation of ART regardless of WHO stage is considered as one way of preventing the occurrence of TB among HIV patients [3].

According to this study, CD4 count was identified as a determinant factor for TB in which patients with a CD4 count of 201–349 cells/mm3 were more likely to develop TB after ART initiation as compared with those with a CD4 count of ≥350 cells/mm3 (AOR: 3.383 95% CI: 1.520, 7.528). It is consistent with a retrospective cohort study conducted in India in which patients with a CD4 count of <200 cells/mm3 were at higher risk to develop TB after ART initiation as compared with those with a CD4 count of >500 cells/mm3 [29]. It is also consistent with a retrospective longitudinal study conducted in Ethiopia in which patients with a CD4 count of >350 cells/mm3 were at lower risk to develop TB [30]. This may be due to the increased risk of developing TB as the result of compromised immune function as HIV preferentially infects activated CD4 cells. CD4 cells are essential for the integrity of antigen-specific humoral and cell-mediated immune response [31]. As CD4 count decreases, the body’s defense ability decreases, and the disease (HIV) progresses to an advanced stage and develops more serious diseases including TB other than mild cutaneous manifestations which develop at the early stage of the disease. WHO and Ethiopian FMoH comprehensive HIV prevention, care, and treatment guideline recommended early initiation of ART after the confirmation of HIV diagnosis regardless of CD4 count for better immunological and clinical improvement as a strategy for the prevention of tuberculosis among HIV patients [3,5].

This study identified the use of IPT as a determinant factor for the development of TB in which patients who didn’t take IPT were more likely to develop TB after ART initiation (AOR: 3.701 95% CI: 2.228, 6.147) which is consistent with a retrospective study conducted in Afar region and at Debre-Markos referral Hospital [9,25]. This is due to the effect of Isoniazid Preventive Therapy in the prevention of reactivation of latent TB infection to active TB disease. Studies supported that using IPT does not increase the risk of developing Isoniazid resistant TB. So, according to the current guideline, the development of Isoniazid resistant TB should not be a concern for providing IPT and it should be initiated after ruling out active TB disease, peripheral neuropathy, active hepatitis, and other contraindications [3]. In contrast, a retrospective cohort study conducted in Tanzania showed that patients who received IPT were at higher risk to develop TB [32]. This may be due to a difference in sample size. The number of total samples involved in the study affects the representativeness of samples and the precision of the data. The larger the sample size is the more representative of the general population and the smaller sample size tends to produce a less accurate estimate and an increased sampling error [33].

According to this study, adherence to ART medicine is identified as a statistically significant determinant factor for the occurrence of TB after ART in which patients who are poorly adherent (AOR: 2.626 95% CI: 1.272, 5.423) or moderately adherent (AOR: 3.455 95% CI: 1.885, 6.335) to ART were more likely to develop TB as compared with those patients who are good adherent. This can be explained by that poor adherence to ART leads to rapid viral replication and worsen immunological and clinical outcomes, increased anti-retroviral drug resistance, and development of treatment failure. Comprehensive HIV prevention, care, and treatment guideline recommended the provision of adherence counseling for people living with HIV at the time of initiation and throughout the course of treatment. The guideline recommended combining viral load monitoring with other approaches for strict assessment of adherence to ART, which is the major cause of drug-resistant and treatment failure [3].

The finding from this study is important to provide information for policymakers, governmental and non-governmental organizations, health professionals, and researchers. Such institutions should consider the findings from this study while developing strategies, policies, and guidelines and during the prioritization of services targeting care and treatment of patients living with HIV. This study is an important input for ART health professionals to build up their knowledge regarding factors that determine the development of TB after initiation of ART. It also helps them to plan for counseling services targeting HIV patients. The possible limitations of this study are social desirability bias and recall bias for questions related to substance use and adherence to ART. In addition to this, using a medical record as a source of information may affect the accuracy of the information.

## Conclusion

This study identified that Poor housing condition like having a kitchen within the living room from environmental factors, having OIs, low CD4 count, and starting ART at the advanced HIV stage (stage III and IV) from immunological and clinical factors were associated with the occurrence of TB after initiation of ART. The use of IPT and being adherent to the prescribed antiretroviral therapy has a protective effect against the development of TB after initiation of ART. Therefore, early screening should be done for TB among HIV patients having such determinant factors identified by this study. Strict evaluation should be done for counseling services regarding IPT use, adherence, and housing condition.

## Supporting information

S1 Data SPSS statistics data. (SAV)
